# Supplementary material for: Task-induced brain state manipulation improves prediction of individual traits
Source: Nat Commun. 2018 Jul 18;9:2807. doi: 10.1038/s41467-018-04920-3 (PMC6052101; doi:10.1038/s41467-018-04920-3)
Supplement: Supplementary file 1 — Supplementary Information [file 41467_2018_4920_MOESM1_ESM.pdf]

Supplementary Information

Task-induced brain state manipulation improves prediction of individual traits

Greene et al.

Supplementary Note 1

Analyses of effects of gF measurement technique

PNC CPM results prove relatively robust to gF measurement technique and measurement-based exclusion criteria (Supplementary Table 2). However, it is unsurprising that when the sample size is decreased (it was effectively halved for the "18-item" and "24-item" analyses), the number of edges that survive thresholding on all iterations of the CPM pipeline is substantially decreased, rendering model performance patterns less stable. For example, at an edge-selection threshold of $P < 0.001$, WM task-based models built using data from only those subjects who completed the 18-item Penn Matrix Reasoning Test included only 41 and 19 edges in the correlated network (CN) and anti-correlated network (AN), respectively, as compared to 305 and 276 edges in the whole-sample CN and AN, respectively. To ensure inclusion of a sufficient number of edges for robust prediction, results of these analyses are presented in Supplementary Table 2 for models generated with an edge-selection threshold of $P < 0.01$.

Sex differences analyses

Sex differences in model performance prove relatively robust to edge-selection threshold (Supplementary Table 10). However, as in the analyses using only those subjects who performed a given version of the Penn Matrix Reasoning Test, the number of edges that survive thresholding when the sample is split by sex is substantially decreased. For example, in the HCP data, the emotion task-based model appears to outperform the WM task-based model in males at an edge-selection threshold of $P < 0.001$, but closer inspection reveals that the emotion task-based CN only included 18 edges, and the AN only included 16 edges, as compared to 89 and 79 edges in the whole-sample CN and AN, respectively, using the same threshold. At all other thresholds tested in the HCP data set, the WM task-based model outperformed the emotion task-based model in males, again suggesting the instability of predictions made by models that include so few edges. Again, to ensure inclusion of a sufficient number of edges for robust prediction, results of the sex difference analyses are reported in the main text using models generated with an edge-selection threshold of $P < 0.01$.

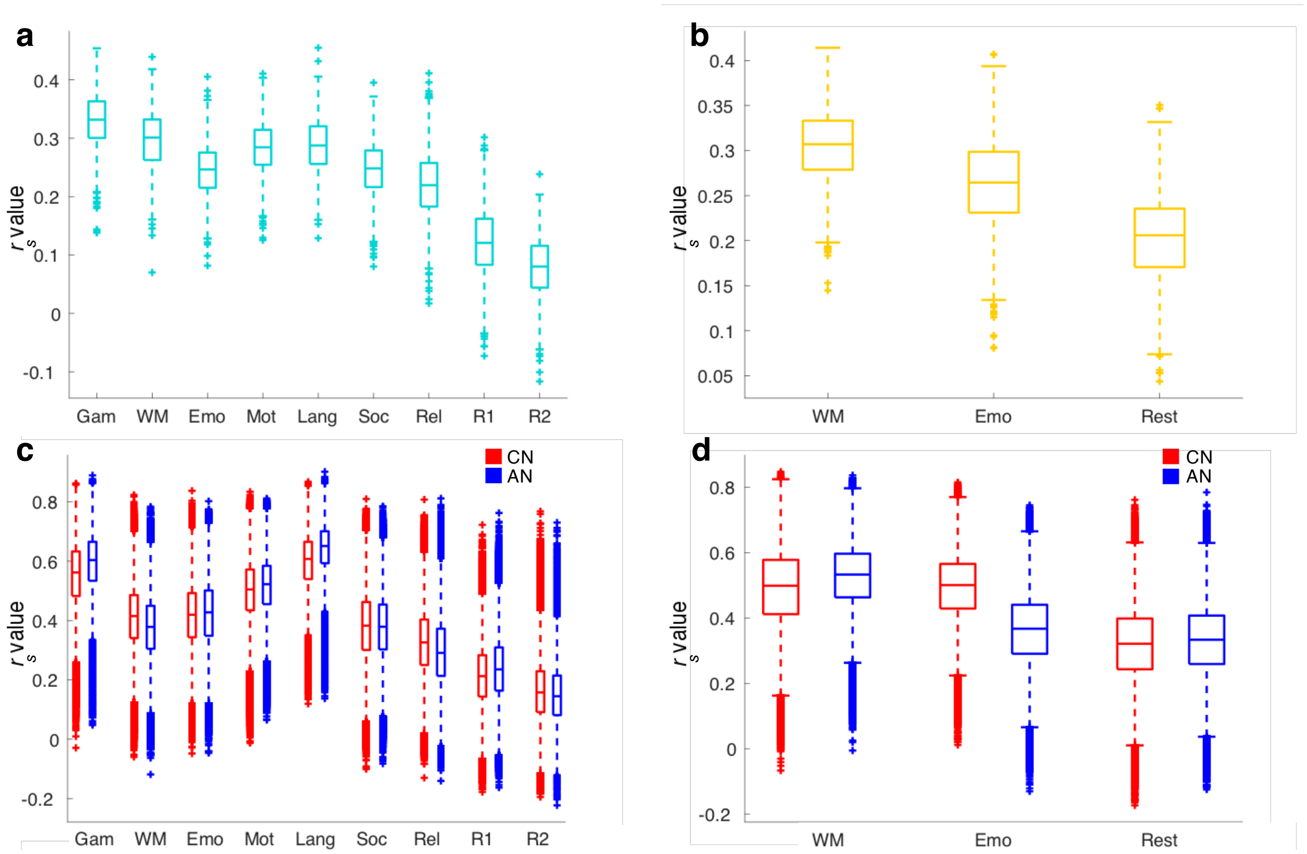

Supplementary Figure 1. Prediction performance (a,b) and model anatomical distribution (c,d) are relatively stable across 1,000 iterations of split-half predictive modeling in both the HCP (a,c) and PNC (b,d) data sets. Models generated using an edge-selection threshold of $P < 0.01$. Results presented as the Spearman's correlation between predicted and true gF (a,b) and between node degree vectors for every pair of iterations. In each boxplot, center line corresponds to the median value, box edges correspond to the 25th and 75th percentiles, and whiskers extend to the most extreme data points not considered outliers. Outliers plotted individually.

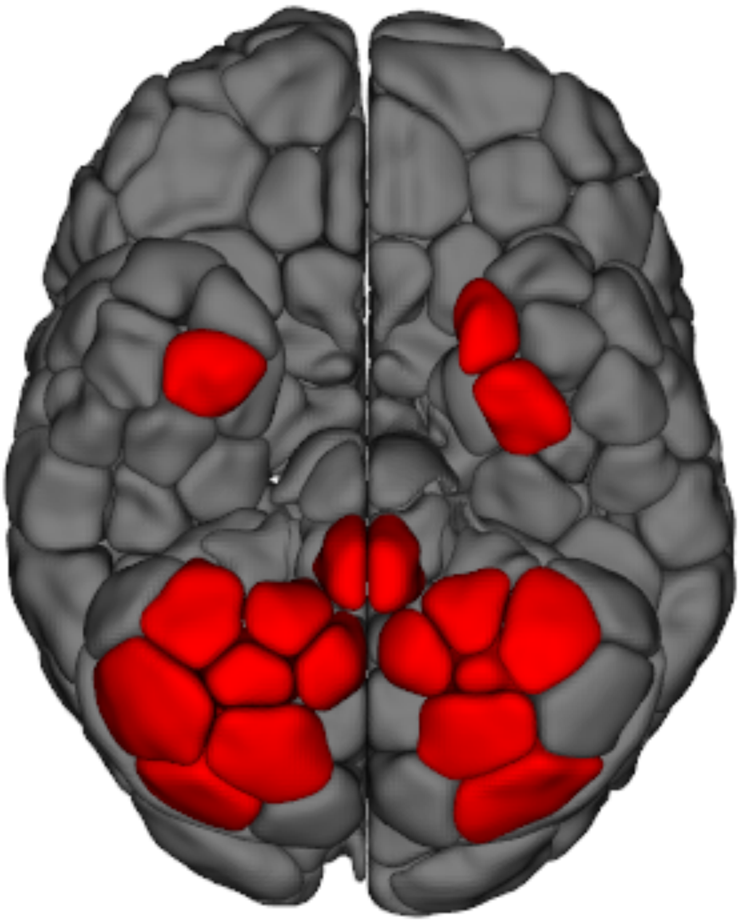

Supplementary Figure 2. Anatomical distribution of nodes with incomplete coverage in the PNC data set.

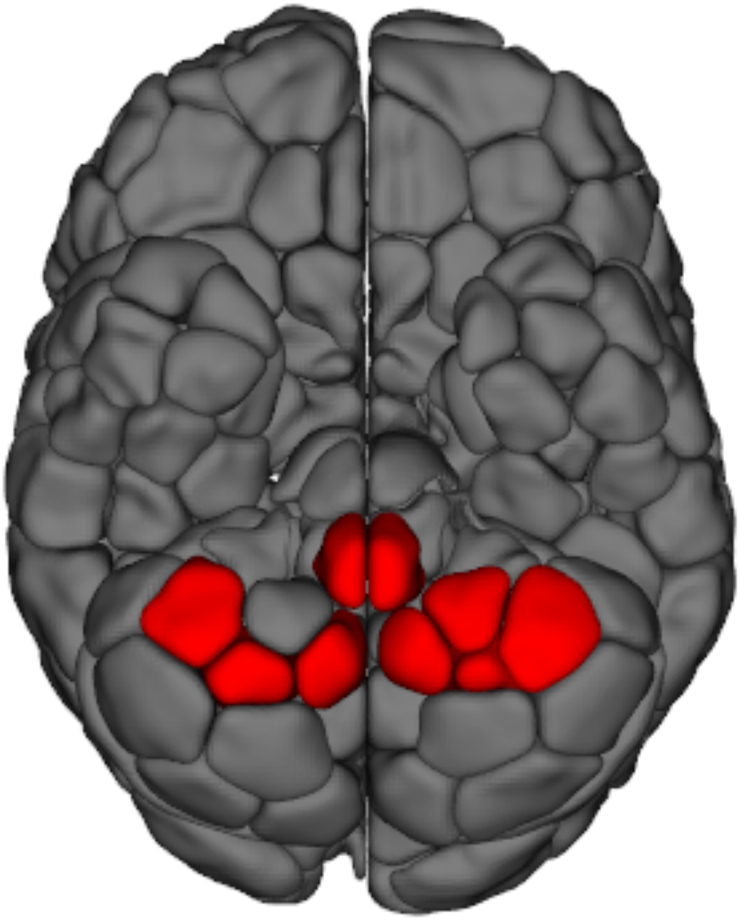

Supplementary Figure 3. Anatomical distribution of nodes with incomplete coverage in the HCP data set.

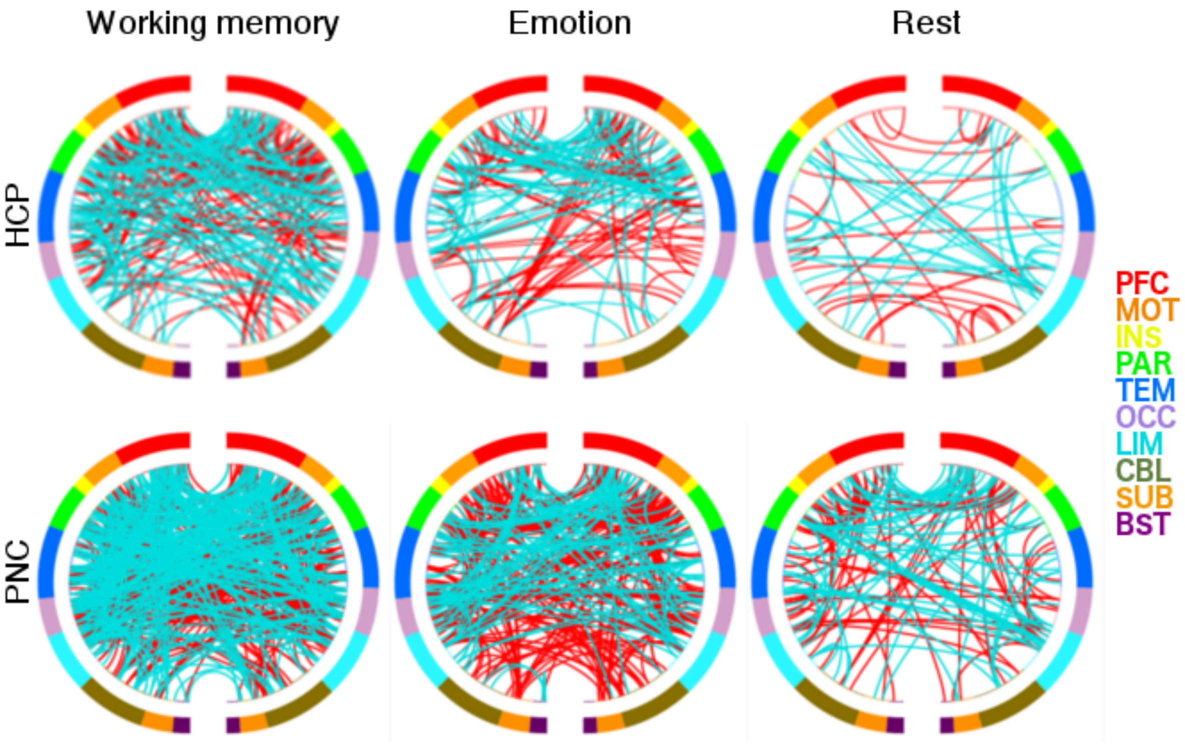

Supplementary Figure 4. Edges selected in each model using an edge-selection threshold of $P < 0.001$. In each circle plot, nodes (inner circle) are grouped anatomically into lobes (outer circle) split into left and right hemispheres. Each line represents one edge between a node pair. Correlated network (CN) edges are displayed in red; anti-correlated network (AN) edges are displayed in blue. PFC, prefrontal cortex; MOT, motor; INS, insula; PAR, parietal lobe; TEM, temporal lobe; OCC, occipital lobe; LIM, limbic system; CBL, cerebellum; SUB, subcortical; BST, brainstem.

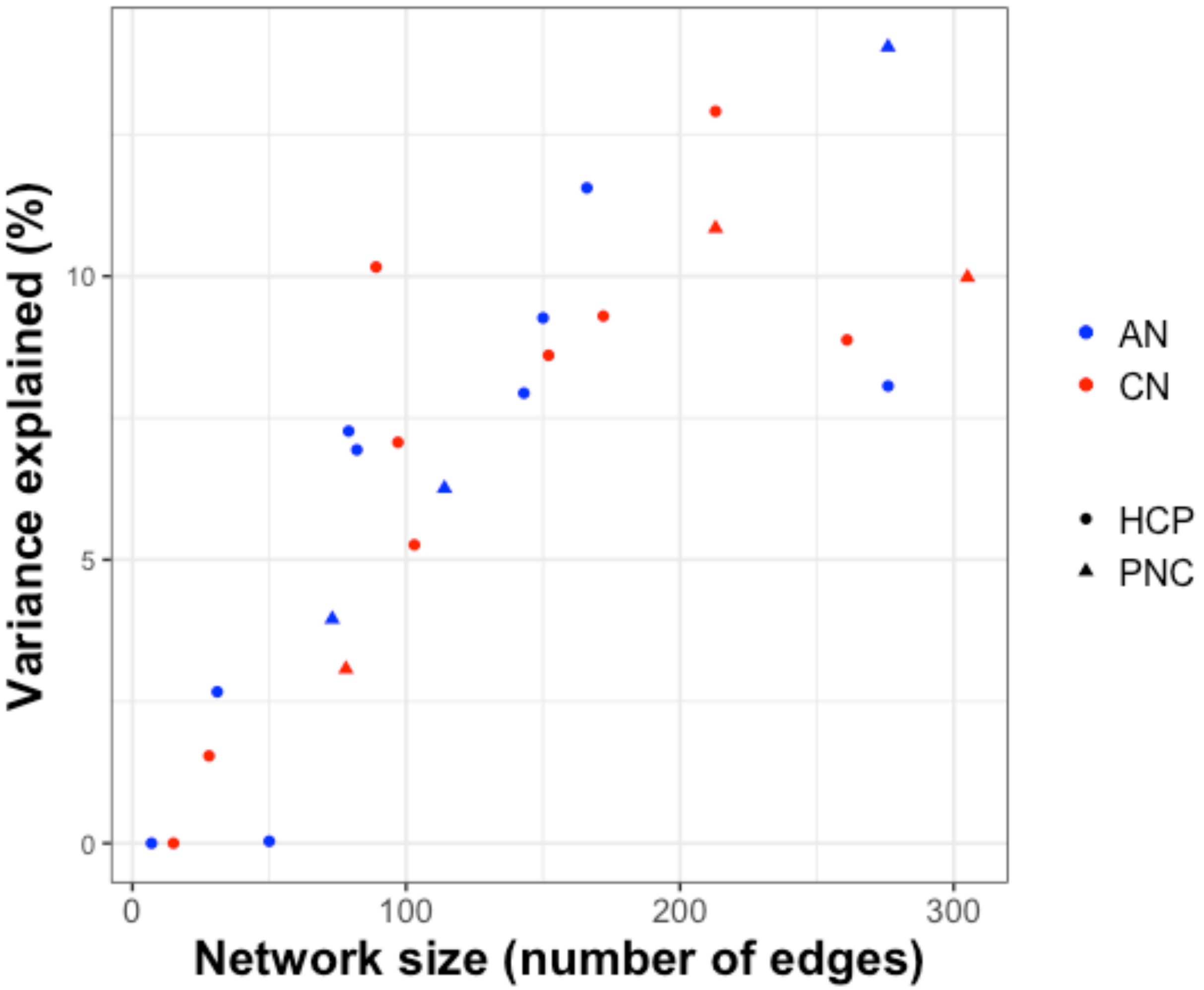

Supplementary Figure 5. Relationship between model size (number of edges selected using an edge-selection threshold of $P < 0.001$) and model performance (plotted as percent gF variance explained) for every AN and CN in the HCP (9 of each, one per condition) and PNC (3 of each) data sets.

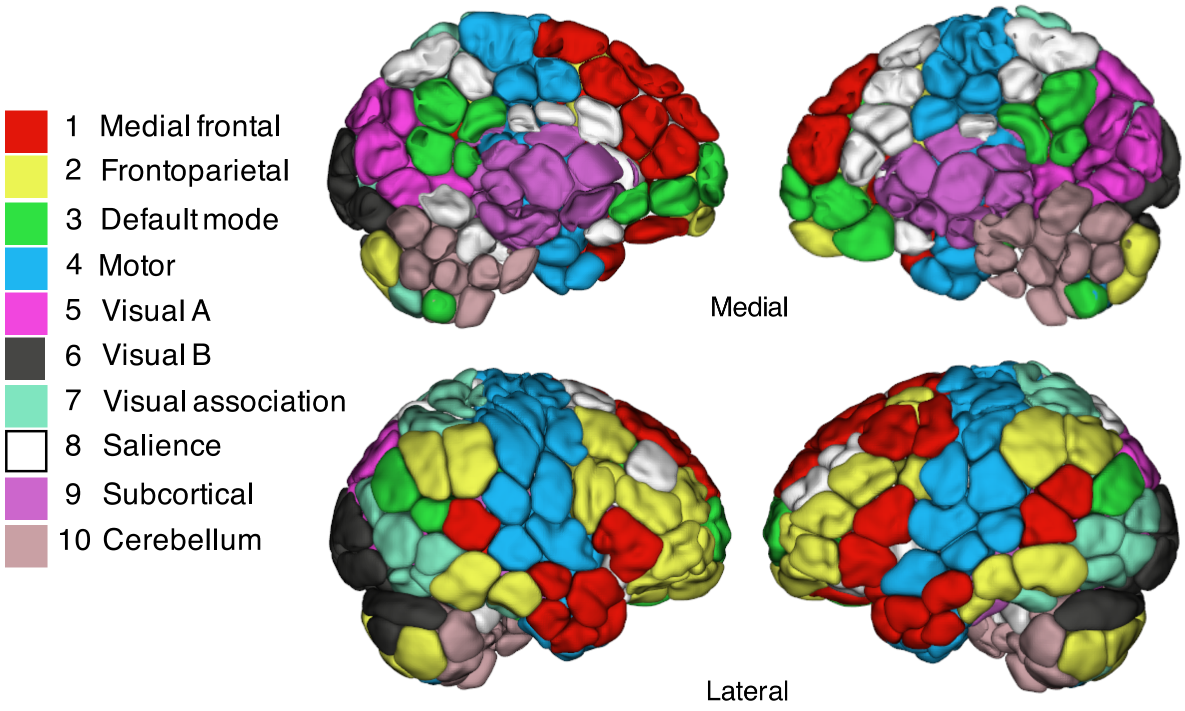

Supplementary Figure 6. Ten canonical networks used for model localization analyses (for a description of how networks were generated, see Methods).

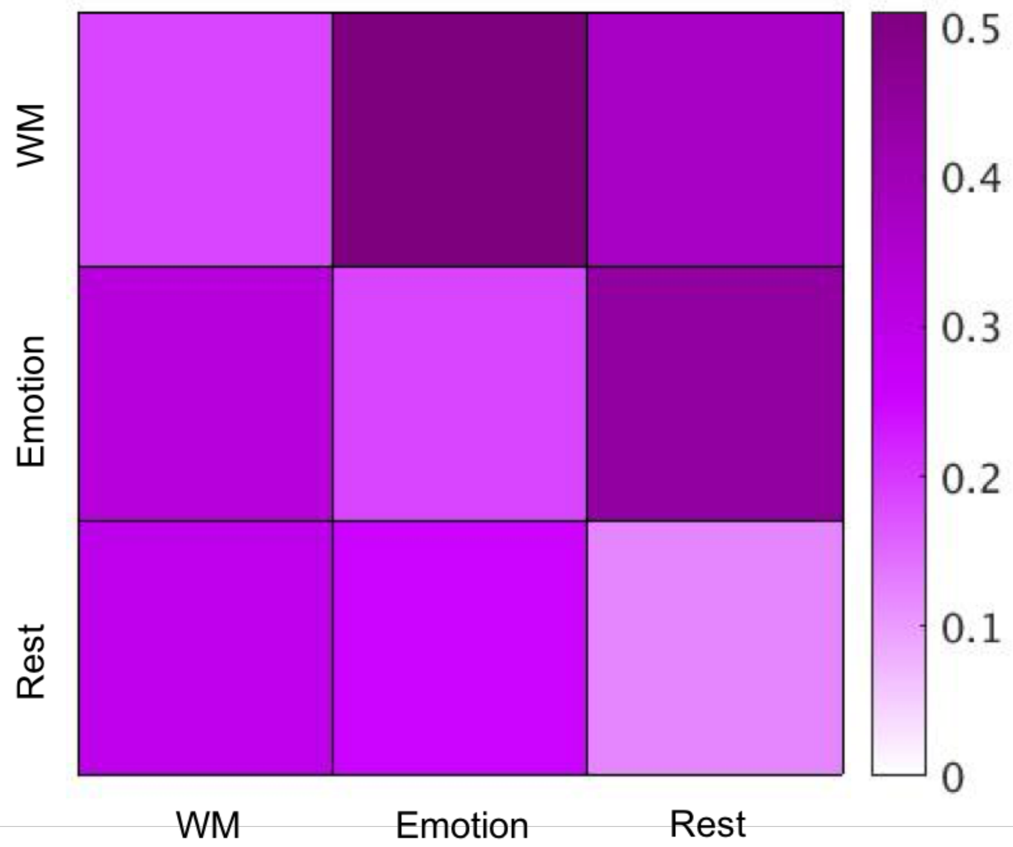

Supplementary Figure 7. Similarity of edges' correlations with gF between conditions, demonstrating substantial overlap between models both within data sets (PNC data: upper triangle; HCP data: bottom triangle) and between data sets (main diagonal). Values indicate Spearman's correlation coefficients.

	Task	Edge-selection threshold						
		$P < 0.001$	$P < 0.005$	$P < 0.01$	Top 1%	Top 2.5%	Top 5%	Top 10%
**HCP**	Gam	12.8	13.2	13.7	13.6	13.4	12.6	11.6
	R1	2.9	3.1	1.7	2.7	2.3	1.8	1.7
	R2	0	1.8	0.3	0.7	2.0	2.0	2.6
	Lang	8.7	8.5	8.5	8.7	8.6	8.5	7.6
	Mot	8.8	8.9	8.2	7.9	8.3	8.7	9.2
	Rel	3.4	6.4	5.5	5.9	6.1	6.5	7.4
	Soc	6.5	8.5	7.7	7.8	8.2	8.0	6.8
	WM	10.6	12.2	11.2	11.9	10.6	10.2	12.4
	Emo	9.8	6.8	8.4	7.2	7.3	7.3	6.9
**PNC**	WM	12.3	10.2	9.7	11.2	9.8	10.6	10.6
	Emo	9.9	9.1	7.7	10.4	7.3	7.8	7.2
	Rest	3.9	5.0	5.9	5.4	5.1	4.7	4.6

Supplementary Table 1. Patterns of CPM results hold across various edge-selection thresholds. In this and all subsequent tables, except where otherwise noted: results reported as percent variance explained ($100r_s^2$); HCP n = 515, PNC n = 571; Gam, gambling task; R1, rest1; R2, rest2; Lang, language processing task; Mot, motor task; Rel, relational processing task; Soc, social cognition task; WM, working memory task; Emo, emotion processing task.

	Task	Pmat CR					Pmat PC
		"V" only (n = 560)	18-item (n = 225)	24-item (n = 335)	Partial correlation (n = 571)	Multilinear regression (n = 571)	n = 571
**PNC**	WM	10.0	7.0	12.2	11.3	12.3	10.8
	Emo	9.1	1.8	7.4	10.8	10	12.1
	Rest	6.6	2.5	7.8	5.6	3.8	5.4

Supplementary Table 2. Patterns of model performance are relatively robust to measurement-based subject exclusion criteria, gF measure, and incorporation of test version into the modeling pipeline. "'V' only," partial correlation-based, multilinear regression, and Pmat PC models generated using an edge-selection threshold of $P < 0.001$; 18-item and 24-item models generated using an edge-selection threshold of $P < 0.01$, given the reduction in sample size (see Supplementary Note 1). Pmat, matrix reasoning test of gF; CR, number correct; PC, percent correct; V, "valid" Pmat score.

	Task	No QC issues (*n* = 475)	r227 only (*n* = 402)	Partial correlation	Multilinear regression
**HCP**	Gam	14.0	10.2	11.3	12.6
	R1	2.6	1.9	3.2	2.8
	R2	0	0	0	0
	Lang	9.3	7.7	8.4	8.5
	Mot	8.2	6.5	9.0	8.6
	Rel	7.3	2.9	4.8	3.3
	Soc	6.2	6.0	6.1	6.2
	WM	11.1	11.6	10.6	10.5
	Emo	9.0	6.8	10.8	9.5

Supplementary Table 3. HCP model performance is relatively unchanged by exclusion of subjects with quality control (QC) issues or for whom fMRI data were reconstructed using the r177 algorithm, or by incorporation of algorithm version into edge-selection (via partial correlation) or model-building (via multilinear regression) steps. Models generated using an edge-selection threshold of $P < 0.001$.

	Task	Partial correlation	Multilinear regression
**HCP**	Gam	11.6	12.7
	R1	3.3	2.9
	R2	0.1	0
	Lang	8.3	8.6
	Mot	6.4	8.6
	Rel	7.9	3.2
	Soc	3.8	6.5
	WM	10.5	10.4
	Emo	7.7	9.7
**PNC**	WM	12.2	12.9
	Emo	9.4	10.3
	Rest	5.0	4.2

Supplementary Table 4. Incorporating head motion into the modeling pipeline does not substantially affect model performance. Models generated using an edge-selection threshold of $P < 0.001$.

	Task	250 node	600 node
**HCP**	Gam	12.2	
	R1	2.4	
	R2	0	
	Lang	9.1	
	Mot	9.0	
	Rel	3.4	
	Soc	6.5	
	WM	10.6	
	Emo	8.4	
**PNC**	WM		11.2
	Emo		8.2
	Rest		4.7

Supplementary Table 5. Model performance is relatively unchanged by increased parcellation resolution ("600 node") and decreased scan coverage ("250 node"). Models generated using an edge-selection threshold of $P < 0.001$.

	Task	Edge-selection threshold		
		$P < 0.01$	$P < 0.005$	$P < 0.001$
HCP	Gam	11.9	12.1	13.8
	R1	4.7	2.7	2.5
	R2	2.3	2.1	0
	Lang	7.6	7.6	8.2
	Mot	9.7	8.4	8.9
	Rel	3.0	6.2	4.5
	Soc	5.3	7.3	5.7
	WM	8.5	8.4	5.9
	Emo	8.4	6.8	9.8
PNC	WM	12.4	11.5	8.8
	Emo	7.9	8.8	7.7
	Rest	5.9	5.0	3.9

Supplementary Table 6. Overall, task-based models again outperform rest-based models when connectivity matrices are generated using only the first 176 time points of all conditions (HCP), or the first 124 time points of all conditions (PNC).

	Task	Edge-selection threshold		
		$P < 0.01$	$P < 0.005$	$P < 0.001$
**HCP**	Gam	13.0	12.9	13.0
	R1	3.4	4.2	4.9
	R2	1.0	0.4	0
	Lang	8.1	8.2	8.4
	Mot	8.3	8.2	8.3
	Rel	4.8	4.8	4.8
	Soc	8.7	8.6	6.8
	WM	11.4	11.3	10.0
	Emo	6.7	6.9	7.0
**PNC**	WM	10.4	11.0	10.8
	Emo	8.8	9.3	9.7
	Rest	4.4	4.3	3.9

Supplementary Table 7. Use of *k*-fold, rather than leave-one-out, cross-validation ($k = 10$) for model training and testing does not substantially change patterns of model performance in either the HCP or PNC data sets.

	Task	Edge-selection threshold		
		$P < 0.01$ ($n = 514$)	$P < 0.005$ ($n = 514$)	$P < 0.001$ ($n = 514$)
**HCP**	Gam	3.0	3.4	3.8
	R1	1.4	1.6	1.6
	R2	1.2	1.6	1.9
	Lang	4.4	4.8	5.5
	Mot	2.7	3.0	4.0
	Rel	3.1	3.6	4.2
	Soc	4.3	4.8	5.9
	WM	3.4	3.8	4.2
	Emo	3.5	2.9	2.8

Supplementary Table 8. Omission of global signal regression decreases model performance overall, but all task-based models again outperform rest-based models.

	Task	CN		AN	
		Right to Left	Left to Right	Right to Left	Left to Right
**PNC**	WM	0.55 (5.0E-11)	0.56 (7.4E-12)	0.57 (8.6E-12)	0.62 (1.4E-14)
	Emotion	0.57 (6.3E-12)	0.54 (1.1E-10)	0.51 (2.5E-9)	0.45 (1.8E-7)
	Rest	0.29 (0.001)	0.31 (3.7E-4)	0.38 (1.3E-5)	0.40 (4.0E-6)
**HCP**	WM	0.32 (2.3E-4)	0.40 (3.2E-6)	0.38 (1.3E-5)	0.37 (1.9E-5)
	Emotion	0.27 (0.002)	0.27 (0.002)	0.32 (2.1E-4)	0.31 (3.3E-4)
	Rest1	0.17 (0.052)	0.19 (0.034)	0.31 (3.4E-4)	0.30 (6.5E-4)

Supplementary Table 9. Degree maps demonstrate substantial bilateral symmetry, as indicated by the Spearman's correlation of node degree across hemispheres. Correlations were performed both between degree of right-hemisphere nodes and their assigned left-hemisphere homologs ("Right to Left"), and between left-hemisphere nodes and their assigned right-hemisphere homologs ("Left to Right") for both the CN and AN. Results reported as $r_s(P)$; models generated using an edge-selection threshold of $P < 0.01$.

	Task	*P* < 0.01 M	*P* < 0.01 F	*P* < 0.005 M	*P* < 0.005 F	*P* < 0.001 M	*P* < 0.001 F	Partial correlation	Multilinear regression
HCP	Gam	14.4	7.6	15.3	7.2	14.8	3.5	12.1	12.7
	R1	0.7 *(82,73)*	0.1 *(69,107)*	0.2 *(43,31)*	0.2 *(36,50)*	4.2 *(8,3)*	2.2 *(7,8)*	2.8	2.6
	R2	1.9 *(108,104)*	0.4 *(111,72)*	1.4 *(49,51)*	0.1 *(62,37)*	0.1 *(4,6)*	0 *(4,8)*	1.8	0
	Lang	6.2	7.9	7.3	8.5	6.0	10.0	10.1	8.5
	Mot	9.0	5.3	8.5	3.4	15.5	3.6	9.0	8.7
	Rel	3.8	5.9	3.6	3.9	2.4	0.3	6.4	3.4
	Soc	14.6	4.2	13.0	3.5	17.4	4.2	5.9	6.3
	WM	20.3 *(334,325)*	0.5 *(174,150)*	16.7 *(200,173)*	3.2 *(95,78)*	11.5 *(59,49)*	3.4 *(28,18)*	10.7	10.4
	Emo	7.3 *(126,122)*	5.9 *(253,253)*	4.8 *(70,69)*	6.5 *(146,149)*	13.7 *(18,16)*	8.9 *(48,39)*	10.2	9.7
PNC	WM	9.7 *(437,404)*	6.3 *(270,266)*	11.3 *(274,233)*	4.3 *(153,155)*	12.0 *(88,80)*	3.7 *(36,40)*	7.7	12.2
	Emo	4.0 *(289,226)*	11.8 *(394,236)*	5.1 *(144,126)*	13.0 *(251,131)*	6.3 *(38,35)*	13.7 *(99,52)*	4.5	10.1
	Rest	5.3 *(262,286)*	3.7 *(147,148)*	4.9 *(142,164)*	4.9 *(81,85)*	4.4 *(28,46)*	7.7 *(27,23)*	1.2	4.0

Supplementary Table 10. Sex differences in model performance are relatively robust to various edge-selection thresholds (illustrative edge counts for shared conditions in parentheses [order: *(CN, AN)*]; see Supplementary Note 1), and incorporation of sex into edge-selection (via partial correlation [with sex and age in the PNC data]) and model-building (via multilinear regression) steps (using an edge-selection threshold of $P < 0.001$) does not substantially improve model performance.

Canonical network	Number of nodes		
	Overall	HCP	PNC
MF	29	29	29
FP	34	34	31
DMN	20	18	18
Motor	50	49	46
Vis A	18	18	18
Vis B	9	9	8
Vis Assoc	18	18	17
Salience	30	30	30
Subcortical	29	29	29
CBL	31	25	24

Supplementary Table 11. Number of nodes in each of the ten canonical networks in total ("Overall"), and after node exclusion for incomplete coverage in each data set. MF, medial frontal; FP, frontoparietal; DMN, default mode network; Vis A, visual A; Vis B, visual B; Vis Assoc, visual association; CBL, cerebellum.
